# Signaling in Effector Lymphocytes: Insights toward Safer Immunotherapy

**DOI:** 10.3389/fimmu.2016.00176

**Published:** 2016-05-12

**Authors:** Kamalakannan Rajasekaran, Matthew J. Riese, Sridhar Rao, Li Wang, Monica S. Thakar, Charles L. Sentman, Subramaniam Malarkannan

**Affiliations:** ^1^Laboratory of Molecular Immunology and Immunotherapy, Blood Research Institute, Milwaukee, WI, USA; ^2^Laboratory of Lymphocyte Biology, Blood Research Institute, Milwaukee, WI, USA; ^3^Department of Medicine, Medical College of Wisconsin, Milwaukee, WI, USA; ^4^Department of Microbiology and Molecular Genetics, Medical College of Wisconsin, Milwaukee, WI, USA; ^5^Laboratory of Stem Cell Transcriptional Regulation, Blood Research Institute, Milwaukee, WI, USA; ^6^Department of Pediatrics, Medical College of Wisconsin, Milwaukee, WI, USA; ^7^Department of Microbiology and Immunology, Center for Synthetic Immunity at the Geisel School of Medicine at Dartmouth, Lebanon, NH, USA

**Keywords:** signaling, NK and T cells, immunotherapy, target molecules, NKG2D

## Abstract

Receptors on T and NK cells systematically propagate highly complex signaling cascades that direct immune effector functions, leading to protective immunity. While extensive studies have delineated hundreds of signaling events that take place upon receptor engagement, the precise molecular mechanism that differentially regulates the induction or repression of a unique effector function is yet to be fully defined. Such knowledge can potentiate the tailoring of signal transductions and transform cancer immunotherapies. Targeted manipulations of signaling cascades can augment one effector function such as antitumor cytotoxicity while contain the overt generation of pro-inflammatory cytokines that contribute to treatment-related toxicity such as “cytokine storm” and “cytokine-release syndrome” or lead to autoimmune diseases. Here, we summarize how individual signaling molecules or nodes may be optimally targeted to permit selective ablation of toxic immune side effects.

## Introduction

Immune responses by NK and T cells control infection and nascent malignancy. Generation of optimal immune responses requires efficient lymphocyte differentiation, proliferation, trafficking, recognition of target antigens, production of inflammatory cytokines, and lysis of infected and tumor cells. Functions of effector lymphocytes must be tightly regulated to prevent generation of uncontrolled immune responses. When they become chronic, uncontrolled immune responses can lead to autoimmune diseases such as rheumatoid arthritis (1), autoimmune vasculitis (2), or encephalomyelitis (3). Recent advances in utilizing synthetic receptors such as chimeric antigen receptors (CARs) have significantly augmented antitumor responses. CAR consist of an antigen-binding domain, linked to a primary signaling domain, such as CD3zeta, and often contain a co-stimulation domain from CD28 or 4-1BB to enhance NK or T cell activation (4). This enhancement also results in a heightened level of inflammatory cytokine and chemokine production. When this inflammatory response is acute, it can lead to “tumor lysis syndrome” (TLS), “cytokine storm” (CS), or “cytokine release syndrome” (CRS), which result in multi-organ failure leading to life-threatening situations.

Tumor lysis syndrome represents rapid death of malignant cells in patients with bulky tumors and release of cellular contents into circulation. This occurs following responsive treatments in patients with tumors that are highly proliferative. In addition, TLS can also occur spontaneously (5, 6). In most cases, kidney functions of the patients are compromised due to the large volume of intracellular contents released into the blood circulation (6, 7).

Cytokine storm also known as hypercytokinemia is the uncontrolled production of proinflammatory cytokines largely due to viral infections (8). Acute lung injury is the most common pathological outcome of CS (9). The cause of CS and the resultant elevated cytokine production is primarily has been associated with the activation of T cells. The central cause of CS is yet to be determined; however, a loss of feedback controls assumed to be the basis. CS can result in tissue and organ damage and at chronic conditions, can result in death.

Cytokine release syndrome is a complex clinical phenomenon characterized by the high activation of immune cells and production of proinflammatory cytokines. CRS can occur during severe infections, graft-versus-host disease, and after treatment with a variety of immunomodulating therapies, such as monoclonal antibodies, bispecific antibodies, or CAR T cells (10–12). Clinical manifestations of CRS include fever, nausea, diarrhea, headaches, confusion, seizure, hypotension, tachycardia, tachypenea, rash, liver alterations, and renal failure. Hours to days after treatment, patients experience symptoms and have elevated amounts of proinflammatory cytokines in their serum, which may include IFN-γ, IL-2, TNF-α, MIP-1, GM-CSF, IL-6, IL-8, or IL-10 (13, 14). In some cases, the CRS appeared in patients 1–4 weeks after infusion of CD19-specific CAR T cells at a time when there was a great CAR T cell expansion (15, 16). The outcome of CRS can be life-threatening, and there have been a number of patient deaths after treatment with novel immunotherapy agents (17). When identified early, CRS can be clinically managed, and treatment for CRS is focused on anti-cytokine therapies, such as corticosteroids and anti-IL-6R mAbs (18, 19). Davila and colleagues have defined five criteria for severe CRS, and Lee et al. describes a grading system and treatment algorithm for CRS (20, 21). Currently, numerous NK cell-based clinical trials are underway to treat hematological and solid tumors (22–36). Pioneering clinical applications have been developed using CAR-transduced primary T cells (20, 37). However, development of CRS following CAR T cell treatment is a major concern (12). Life-threatening CRS is an impediment to the clinical utilization and curative efficacy of CAR therapies (18). The identification of a unique signaling pathway that regulates inflammation may help to reduce toxicity and is of high clinical relevance. Therefore, understanding the molecular basis of signal transduction and subsequent regulation of effector functions within lymphocytes has the potential to limit CRS mediated by T cells or NK cells transduced with CARs or after treatment with other immunotherapeutic agents.

**Figure 1:**
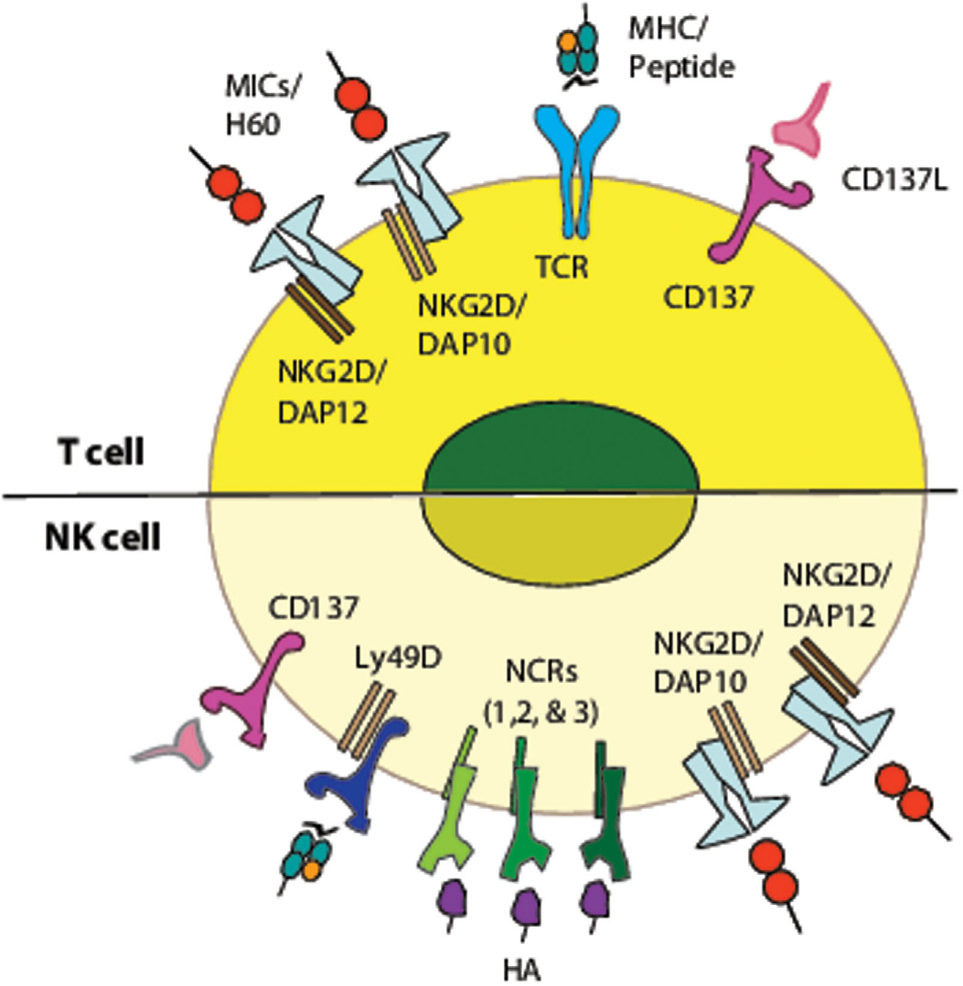
**Activating and co-stimulatory receptors in T and NK cells and their corresponding ligands**. Schematic representation of activating receptors on T cells and NK cells. While T cell receptor (TCR) functions as the primary receptor on T cells, NKG2D and CD137 function as co-stimulatory receptors along with CD28. Whereas, in NK cells, NKG2D, CD137, LY49D, NCR1, and 2B4 function as independent activating receptors and a cumulative effect of their engagement determines the final outcome of NK cell effector functions. The differences in the cytoplasmic domain of these receptors contribute to the interaction of various adapter molecules. These differences govern the signaling cascade that is engaged downstream of these receptors.

NK cells and cytotoxic T cells utilize multiple components of similar signal transduction machineries, but differ in the mode they utilize to activate these signaling components. Unique clonotypic T cell receptors (TCR) equip T cells with exquisite antigen specificity and functions as the most important driver of their activation (Figure 1). In contrast, NK cells integrate signals from a variety of activating and inhibitory receptors in a hierarchical manner to establish an activation state (Figure 1). Conserved non-variant receptors expressed on NK cells recognize “*induced-self*” (NKG2D), pathogen-derived ligands (NCR1), or the yet to be defined mechanism that governs “*missing-self*” (38). Additional classes of receptors (CD16, CD244, Ly49s, and KIRs) are also important in mediating NK cell effector functions. NKG2D and CD137 function as independent activation receptor in NK cells while they play an important co-stimulatory role in T cells (39).

Two of the most important functions of cytotoxic T cells and NK cells are target cell killing and production of pro-inflammatory cytokines. The latter forms a major basis for TLS, CS, and in particular CRS. The qualitative differences in the temporal kinetics and duration of cytotoxicity and pro-inflammatory cytokine production suggest how these might be differentially regulated at the molecular level (40). Here, we summarize membrane proximal and intermediate signal transductions that govern NK cell or T cell activation and how shared or unique signaling molecules elicit specific effector functions. Defining these molecular regulations will help tailor effector functions of T and NK cells in immunotherapeutic strategies.

## Membrane Proximal Signaling

### Src Family Kinases: Lck, Fyn, and Lyn

**Figure 2:**
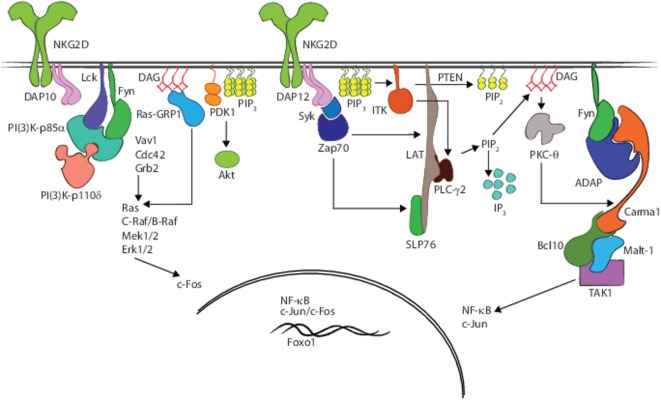
**Receptor interacting and nucleating signaling molecules that regulate the effector functions**. A graphical rendering of membrane proximal signaling events and resultant involvement of scaffold proteins, adapter molecules, and second messengers that are critical for eliciting effector functions such as cytotoxicity and proinflammatory cytokine production following NKG2D-mediated activation in NK cells.

Tyrosine phosphorylation of substrates by Src-family tyrosine kinases represents the first step in NK or T cell activation. Src family members are expressed in numerous tissues and play a vital role in all hematopoietic cell types. Lck, one of the Src members, is central to signal transduction downstream of the TCR and NK cell-activating receptors. Following receptor–ligand interaction, conformational changes take place in the intracellular domains of adapters, such as CD3ζ, that expose ITAMs (*I*mmune *T*yrosine *A*ctivation *M*otifs) for Lck, which localizes to the site of receptor activation through constitutive/induced interaction with co-stimulatory receptors, such as CD4 or CD8 in T cells; CD137 (4-1BB) or NK1.1 (NKR-P1) in NK cells, *via* its Cys–X–Cys–Pro domain; or an inducible interaction with NKG2D in NK cells (41–43). Best-characterized downstream targets of Lck are Syk and Zap-70 (Figure 2). Other imputed interactions of Lck include possible binding to DAP10 and DAP12 adapter proteins (44), and binding to the inhibitory cell surface phosphatase CD45, an interaction that may physically sequester CD45 from TCR and its downstream signaling events (45, 46).

Lck plays a complex role in NK cell signal transduction. Germ-line deletion of Lck results in NK cells with normal development and capacity for activation after stimulation with poly (I:C) or Interleukin (IL)-2 (47). In contrast, either inhibition or knockdown of Lck resulted in significant reductions in NKG2D- and CD137-mediated cytotoxicity and cytokine production in NK cells (Table 1), but no change in the cytokine production mediated by IL-12 and IL-18 stimulation (48). This suggests selective utilization of Lck playing a dominant role downstream of some, but not all activating receptors.

**Table 1:** **Critical signaling molecules that regulate the development, cytotoxicity, or cytokine production from NK cells**.

Signaling protein	Function	Method	Development	Cytotoxicity	Inflammatory cytokines	Our publication
Lck	Membrane proximal kinase	siRNA/pharmacological inhibitor	Not applicable	Reduced	Reduced	
Fyn	Membrane proximal kinase	Knockout	Unknown	Reduced	significantly increased	Rajasekaran et al. (47)
LAT	Scaffold protein	siRNA	Not applicable	Reduced	Reduced	
PLC-γ1	Second messenger generation	Knockout and gene reconstituion	Partial defect in development	Not affected	Not affected	Regunathan et al. (49)
PLC-γ2	Second messenger generation	Knockout and gene preconstituion	Increased NK precursors, Impaired terminal maturation	Significantly impaired	Significantly impaired	
PI3K-p85α	Regulatory subunit of PI3K	Knockout	Impaired terminal maturation, Decreased NK cells	Significantly impaired	Significantly impaired	Awasthi et al. (50)
PI3K-p110δ	Catalytic subunit of PI3K	Knockin	Impaired terminal maturation, decreased NK cells	Significantly impaired	Significantly impaired	Guo et al. (51)
Rap1a	Small GTPase	Knockout	Not affected	Not affected	Not affected	Awasthi et al. (50)
Rap1b	Small GTPase	Knockout	Not affected	Not affected	Significantly impaired	
Carma1	CARD domain-containing scaffold	Knockout/CARD domain deletion	Not affected	Moderately impaired	Significantly impaired	Rajasekaran et al. (48, 52)
Bcl10	CARD domain-containing scaffold	Knockout	Not affected	Moderately impaired	Significantly impaired	Malarkannan et al. (53)
TAK1	MAPKKK	Conditional knockout	Not applicable	Moderately impaired	Significantly impaired	Rajasekaran et al. (48, 52)
ADAP	Scaffold protein	Knockout	Unknown	Not affected	Significantly impaired	Rajasekaran et al. (48)

Fyn is another well-characterized Src family tyrosine kinase with a molecular weight of 59 kDa (54). Although the target substrates of Lck and Fyn appear redundant, it is evident that they play non-overlapping roles (55, 56). For instance, mice deficient of Fyn demonstrate minor impairments in T cell development, while lack of Lck results in a significant block in their development (55). T cells deficient in both Lck and Fyn demonstrate a complete block in T cell development (57). NK cells deficient in Fyn demonstrate a proliferative defect with only a modest enhancement observed with concurrent deficiency of Lck (58). Additionally, NK cells also utilize other Src family kinases such as Src itself, Lyn, and Fgr, although the relative importance of these kinases is uncertain (59–61).

In T cells, Lck has been shown to phosphorylate Fyn (62, 63) following ligand-induced TCR–CD4 co-aggregation. Fyn phosphorylation by Lck does not require other components of the TCR signaling apparatus, since ectopic expression of Fyn and Lck in NIH 3T3 fibroblast results in Fyn phosphorylation in a manner dependent on Lck kinase activity (63). Like Lck, Fyn subsequently phosphorylates Syk family members such as Zap-70 (64). While deficiency of *Fyn* is insufficient to significantly affect downstream TCR signaling events such as activation of Zap-70, LAT, and PLC-γ1, concurrent loss of Fyn and abrogation of Lck-CD4-TCR complex formation results in impaired downstream signals (65). This suggests that function of Fyn is largely redundant with that of Lck, but may play a more specialized role in facilitating TCR signaling.

Apart from its role in activation, Fyn may also play a suppressive role in T cells (66) and NK cells (48). For instance, activation of *Fyn*^−/−^ NK cells *via* NKG2D or CD137 results in significantly elevated levels of proinflammatory cytokine and chemokine production (Table 1) compared to that of wild type (WT) (48), or following Ly49D cross linking (67). Additionally, co-culture of WT or *Fyn*^−/−^ NK cells with target cells such as H60- or CD137L-expressing EL4 or RMA/S and YAC1 resulted in significantly reduced production of proinflammatory cytokines and chemokines in *Fyn*^−/−^ NK cells (48, 58). These findings indicate a high level of complexity in Src family kinase activation that reflects components of redundancy and, perhaps, antagonism in lymphocyte activation.

Distinct from Lck and Fyn, the third Src-kinase family member Lyn acts predominantly as a negative regulator of lymphocyte functions (68). Hyperactivity associated with increased basal and inducible PI(3)K activity in *Lyn*^−/−^ B cells indicates that it may impose inhibitory regulation of other Src-family kinases (69). Consistent with this observation, *Lyn*^−/−^ mice develop autoimmunity resulting, in part, from hyperactive and increased absolute numbers of B and T cells (70). The mechanism underlying these changes results from hypophosphorylation of PAG/Cbp (phosphoprotein associated with glycosphingolipid-enriched microdomains/Csk-binding protein) and increased activity of Fyn (69); however, the direct substrates of Lyn responsible for these changes are yet to be determined. Further studies are needed to delineate the exact mechanism whereby Lyn regulates other signaling molecules.

### PI(3)-Kinase

Activation of Src family kinases in NK cells is crucial for the stimulation of multiple downstream signaling events. This includes PI(3)-Kinase [PI(3)K] that are comprised of regulatory (p85α, p55α, p50α, p85β, and p55γ) and catalytic (p110α, p110β, p110γ, and p110δ) subunits that function to generate the second messenger phosphoinositol (3,4,5) trisphosphate [PI(3,4,5)P_3_] (71). Absence of regulatory or catalytic subunits of PI(3)K significantly impairs the development and functions of lymphocytes (50, 51, 72, 73). PI(3)K-p85α requires membrane localization for optimal activation, and can be recruited to the membrane through multiple mechanisms including binding to a YXXM motif present in the cytoplasmic tail of co-stimulatory receptors (CD19 and CD28) (74, 75), inhibitory receptors (CTLA4), signaling protein (Grb2), or adapter protein (DAP10) (76, 77). In addition, CD137 (4-1BB) recruits PI(3)K-p85α *via* an association with Lck and Fyn (48). In T cells, activation of PI(3)K and generation of PIP_3_ is largely driven by ligation of co-stimulatory receptors, such as CD28 (78). Once localized to the inner leaflet of plasma membrane, using their SH3 domains, Lck and Fyn can bind to the N-terminal proline-rich region (PRR) of the PI(3)K-p85α subunit (79), leading to the phosphorylation of the p85 and recruitment of catalytic p110 isoforms (76). Thus, Src family kinases, through high-affinity interaction with PI(3)K-p85α, function as a critical link between an activation receptor and generation of PIP_3_ (56, 76, 79).

Once generated, PIP_3_ binds and anchors multiple signaling molecules to the plasma membrane including Akt permitting its subsequent phosphorylation by phosphoinositide-dependent kinase-1 (PDK-1) at Thr^308^ (Figure 2). PIP_3_-dependent signaling is terminated through the dephosphorylation of PIP_3_ by lipid phosphatase PTEN that produces PI(4,5)P_2_ (80, 81) or SHIP-1 that generates PI(3,4)P_2_ (82) (Figure 2). Though the phosphoinositides generated by PTEN and SHIP-1 are incapable of binding to Akt, the reduction in the Ser^473^ phosphorylation of Akt in the bone marrow-derived mast cells obtained from SHIP-1 knockout mice and a concomitant reduction in its kinase activity demonstrate the relevance of these signaling intermediates (83). In general, PI(3)K has pro-growth effects in T and NK cells, and PTEN has growth suppressive effects such that deletion of PTEN from T_H_1 cells results in increased proliferation and Akt phosphorylation when compared with WT cells (84).

In NK cells, activation of PI(3)K is crucial for effector functions (50, 72, 73, 85–87). NK cells that lack p85α*^−^* (50) or expressing a mutant form of p110δ (p110δ^D910A^) that lack kinase activity (51) are impaired in both cytotoxicity and cytokine production (Table 1). Moreover, perforin-dependent cryptococcal microbicidal activity of NK cells requires PI(3)K-mediated activation of ERK1/2 (88), and cytotoxic granule mobilization in NK cell is dependent on the PI(3)K–Rac–PAK–ERK1/2 pathway (89). These findings indicate that PI(3)K is a critical proximal signaling module responsible for regulating multiple effector functions.

Deletion of PI(3)K subunits leads to hypo-responsive effector T cells, whereas deletion of E3 ubiquitin ligases that induce degradation of p85 subunits are functionally hyper-responsive (90, 91). Interestingly, modulation of various subunits of PI(3)K has variable impacts on different T cell subsets. For instance, deletion of p110δ in T cells results in enhanced tumor clearance and relative increases in cytotoxic T cell activity, largely because any changes in cytotoxic T cells are countered by profound inactivation of immunosuppressive regulatory T cells (92).

### The Signalosome: Contribution of LAT and SLP-76

Syk family kinases phosphorylate adapter proteins that nucleate signal transduction complexes. In T cells, Zap70 phosphorylates LAT (Linker of activated T cells) and SLP-76 (SH2 domain-containing leukocyte protein of 76 kDa) (Figure 2). LAT and SLP-76 subsequently serve as a scaffold to bind proteins that mediate downstream signals including GADS and Grb2 to LAT, and Itk, adhesion and degranulation promoting adapter protein (ADAP), Vav1, and PLC-γ1 to SLP-76 (93–97). Despite the lack of enzymatic activity, the presence of LAT and SLP-76 are obligatory during T cell development, since *LAT^−/−^* or *SLP-76^−/−^* lack mature T cells (98, 99).

Role of LAT in signaling in NK cells is not clearly defined. NK cells deficient in LAT alone or LAT and NTAL displayed efficient cytotoxicity against all target cell lines (100), but decreased capacity to generate IFN-γ following co-culture with some (e.g., Rae1β^+^ B16 cells) but no other (e.g., YAC-1 or RMA/S cells) target cells (100). These results suggest that LAT is required (Table 1) for signal transduction in NK cells downstream of “*induced-self*” receptors, but not receptors that facilitate activation from “*missing-self*” or “*non-self*.” Similarly, NK cells from *LAT*^−/−^ mice demonstrated normal development and cytotoxicity (98), likely because of compensation from other scaffolds including SLP-76 (Table 1) (101). Therefore, the requirement of LAT is limited to the generation of pro-inflammatory cytokines and is not required for cytotoxicity. Interestingly, recent data suggest that SLP-76 is required for efficient IFN-γ production and cytotoxicity in NK cells in a manner independent of LAT (102), indicating that T and NK cells exploit these adapter molecules in unique ways to coordinate effector functions (Figure 2).

## Intermediate Signaling Events in Cytotoxic T and NK Cell Lymphocytes

### Second Messengers: DAG and IP3

One event crucial to NK and T cell activation is the localization and activation of PLC-γ molecules into the adapter scaffold complexes (103). LAT, anchored to the plasma membrane, serves as a docking site for PLC-γ. While PI(3,4,5)P_3_ is required for the activation of PLC-γ, action of PTEN on the former leads to the generation of PI(4,5)P_2_ permitting the cleavage of membrane-bound PI(4,5)P_2_ by PLC-γ into the second messengers diacylglycerol (DAG) and IP_3_. Whereas DAG, a membrane signaling lipid, binds and activates RasGRP1 (a Ras-activating molecule) and PKC-θ, soluble IP_3_ binds to proteins that facilitate calcium flux from the endoplasmic reticulum (Figure 2). NK cells and T cells utilize different isoforms of PLC-γ in hydrolysis of PIP_2_; T cells primarily utilize PLC-γ1, whereas NK cells predominantly utilize PLC-γ2 (49, 104–108). Although these cell-specific differences partially result from alterations in protein expression levels of individual isoforms (109), overexpression of PLC-γ1 in NK cells can only partially compensate for PLC-γ2 deficiency in NK cells, for instance, restoring the terminal maturation of NK cells, but failing to fully restore impaired cytotoxicity and cytokine production (Table 1) (49). Further dissection of the defects in NK cell-mediated cytotoxicity in the *Plc-γ2^−/−^* NK cells revealed that NK cells were capable of forming conjugates with target cells and developing Microtubule organizing center (MTOC) polarization, but that calcium mobilization and resulting signaling events were compromised (108). In T cells, in addition to LAT, a unique complex containing SLP-76 and the Tec family kinase Itk (IL-2 inducible kinase) are required for PLC-γ activation (110). While phosphorylation of at least three tyrosines at the N-terminus of SLP-76 by Zap70 appear essential for efficient signal transduction, phosphorylation at Y^145^ appears to be most important for recruitment and activation of Itk, and the subsequent phosphorylation and activation of PLC-γ1 (111) (Figure 2). Mutation of SLP-76 at Y^145^, in general, phenocopies the loss of Itk (112), likely because other Tec family kinases, such as Rlk, can only partially compensate for the lack of Itk-mediated PLC-γ1 activation (113). Thus, apart from binding LAT, PLC-γ1 requires interaction with multiple components of the signalosome in order to optimally coordinate enzymatic activity (94).

Once activated, PLC-γ1 generates IP_3_ and DAG, allowing DAG to bind RasGRP1 to facilitate activation of Ras in T cells (114), along with at least three isoforms of PKC, including PKC-ϵ, PKC-ŋ, and PKC-θ (115). Whereas PKC-ϵ and PKC-ŋ serve in a redundant manner to recruit PKC-θ to the immune synapse (115, 116), the role of PKC-θ is obligatory for initiation of downstream signaling events such as NF-κB activation. The localization of DAG is crucial to its ability to mediate signaling and cytoskeletal changes such as dynein-mediated MTOC orientation and T cell polarization (116).

### Second Messenger to PKC-θ

Apart from binding to DAG, PKC-θ is also regulated by multiple other factors. PKC-θ exists as an inactive high-molecular disulfide-linked complex in naive T cells. During T cell activation, PKC-θ is gradually reduced from a 85 kDa inactive form to a 82 kDa active form by two major redox regulators, glutathione and thioredoxin (117), thus implicating the intracellular redox state as another node for PKC-θ regulation. Further, a C2 phosphotyrosine-binding domain also appears important for optimizing activity of PKC-θ (118). In T cells, efficient PKC-θ activity also requires phospohorylation downstream of PDK1, a PIP_3_-regulated kinase, either directly through PDK1 or through an intermediary serine/threonine kinase (119), indicating the role of PI(3)K and thereby a requirement for co-stimulatory receptor ligation for optimal PKC-θ activation (Figure 2).

Activated PKC-θ is required for efficient *in vivo* function of NK cells, since *PKC-θ^−/−^* mice are unable to eliminate tumor cells lacking expression of MHC-I, secondary to reduced NK cell intra-tumoral migration and decreased NK cell activation (120). Similarly, *PKC-θ^−/−^* NK cells demonstrate reduced cytotoxicity after treatment with Toll receptor agonists, such as poly I:C (121, 122) and reduced cytokine production after stimulation through NK1.1 or Ly49D (123). Though the signaling events responsible for the phenotype of *PKC-θ^−/−^* NK cells has undergone limited analysis, it appears that sustained Erk1/2 and Jnk1/2 activation is abrogated, resulting in decreased nuclear translocation of AP-1 and NFAT (123). Intriguingly, the activation of NF-κB and degradation of IκBα does not appear to be impacted by loss of PKC-θ in NK cells (123), in contrast to T cells in which loss of PKC-θ results in major defects in NFκB signaling (124), leading to abrogation of TCR-induced IL-2 production and proliferation (125).

## Unique Signaling That Regulate Selective Effector Functions

**Figure 3:**
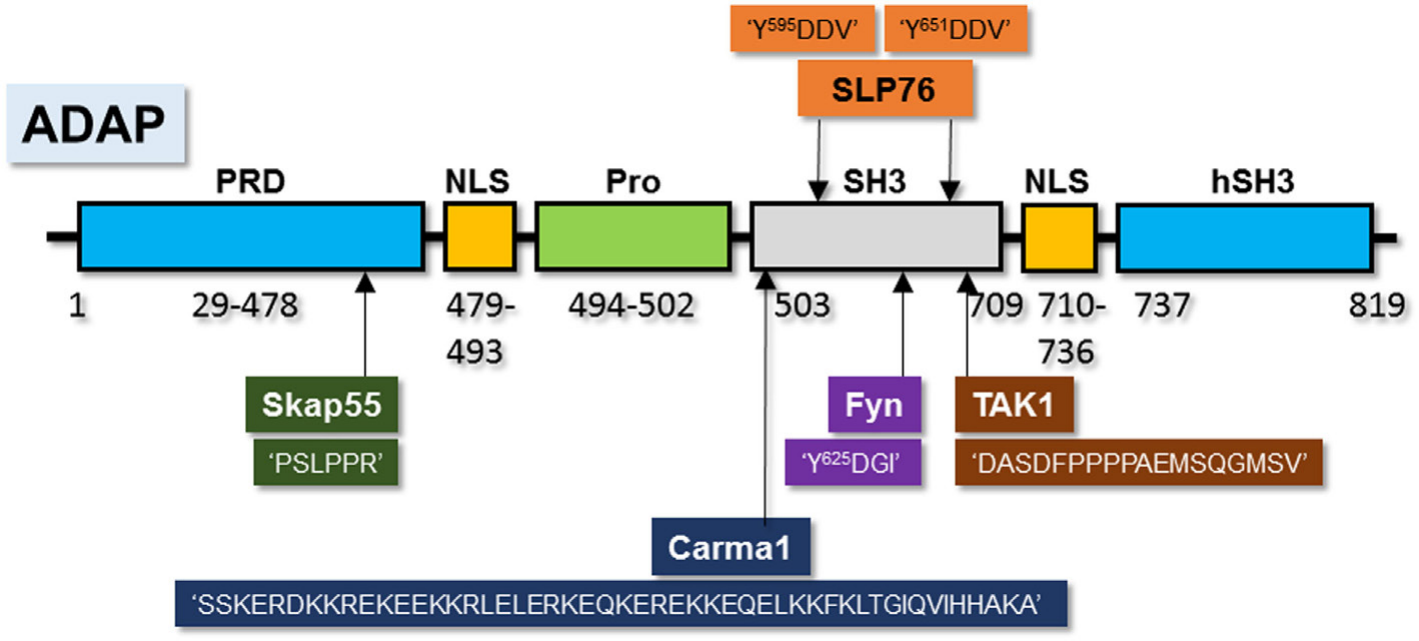
**Unique amino acid motifs in ADAP scaffold facilitate its interactions with multiple-binding partners**. Pictorial depiction of ADAP protein and its amino acid sequences (or motifs) that are required to interact with Fyn, Carma1, TAK1, SLP76, and SKAP55. Amino acid sequences within ‘’ are from ADAP with the name of the interacting partner listed above or below this sequence.

Our recent work has shown that “ADAP” serves as a node of signaling divergence and it could be manipulated to differentially limit unique effector responses. ADAP [Fyn-binding protein (Fyb)] was first identified in T cells as a 120/130 kDa component of the TCR–CD3ζ–p59FynT activation (126). ADAP binds to the SH2 domain of Fyn in a specific manner, such that other SH2-domain-containing Src family kinases, such as Lck, do not (127). Fyb was renamed ADAP in consideration of the specific functional defects identified in lymphocytes from mice deficient of this protein (128). ADAP utilizes several domains to facilitate binding with partner proteins (Figure 3) (129–131) and is known to undergo phosphorylation following TCR ligation, an event that likely contributes to its protein–protein interactions with SLP-76, Nck1, and Nck2 (132). ADAP also binds to the monomeric GTPase Rap1, contributing to its localization at the plasma membrane (133). Interaction of the SH2 domain of SLP-76 with ADAP takes place at three specific sites within ADAP, permitting oligomerization and the formation of SLP-76-based microclusters (134) (Figure 3). ADAP also contains SH3 (hSH3) domains that bind to phosphoinositides that likely contribute to its role in cellular migration, cytokine production, and integrin activation (135).

In NK and effector T cells, ADAP has been shown to play important roles in both ITAM-dependent signaling pathways, and activation of β1 and β2 integrins such as LFA1 (134 and 147). In T cells, loss of ADAP results in a maturation block of double-positive thymocytes resulting from alterations in both positive and negative selection of developing T cells (136). Peripheral T cells that manage to emigrate from the thymus are also impaired as evidenced by reduced tissue rejection in heart (137) and intestinal allografts in *ADAP^−/−^* mice (138), and significant amelioration of pathology in Experimental Autoimmune Encephalitis (EAE) (139). Loss of ADAP in T cells further decreases their proliferation and cytokine production efficiency in response to limiting antigen doses (140). Crucially, ADAP also plays a role in integrin activation of T and NK cells (141). Integrins are components of cell–cell interactions that are required for extravasation and tissue localization of lymphocytes to sites of infection. In resting cells, integrins bind weakly to ligands, such as ICAM1, but in activated cells, integrins are conformationally altered to bind ligands with high affinity. These conformational changes in integrins are due to “inside-out” signaling in contrast to “outside-in” signaling transduced by integrins themselves after ligand binding (142–144). In T cells, the chemokine receptor CCR7 induces activation of the LFA-1 through a mechanism that requires binding of ADAP, and an additional adapter molecule, SKAP55, to LFA-1 (145). Studies using ADAP/SKAP55 chimeric proteins in *Adap^−/−^* T cells identified distinct roles for ADAP in facilitating NF-κB signaling and LFA-1 activation. Expression of a SKAP55/ADAP chimeric molecule in *Adap^−/−^* T cells was sufficient and necessary for integrin function and NF-κB activation (146–148), whereas expression of a chimeric molecule with a point mutation in the PH-domain of SKAP55 permitted restoration of NF-κB activation in NK cells but not integrin function (148). It is currently thought that recruitment of ADAP to LFA1 complexes through the PH-domain of SKAP55 restricts the ability of ADAP to interact with the CBM signalosome and to activate NF-κB signal transduction (148).

Stimulation through TCR and CD28 utilizes ADAP to facilitate signaling downstream of the Carma1–Bcl10–Malt1 (CBM) complex, which leads to phosphorylation and degradation of IκBα and nuclear translocation of NF-κB (149). While the molecular mechanism whereby ADAP regulates the formation of the CBM have not been fully elucidated, the essential function of ADAP in linking CBM *via* Carma1 to PKC-θ is well documented (136). NF-κB, which is sequestered in the cytosol through binding to IκBα, translocates into the nucleus (136). Carma1 and Bcl10 play an obligatory role in the nuclear translocation of NF-kB following activation of NK cells through NK1.1, Ly49D, NKG2D, and CD137 (52, 150). In addition to interactions with Carma1, ADAP also recruit TAK1, which facilitates phosphorylation of IKK alpha and beta (149), components of NF-κB signaling pathway. In NK cells, ADAP contributes to CBM complex formation in response to ITAM-containing receptors such as NK1.1, Ly49D, Ly49H, and response to activation through NKG2D, NCR1, and CD137 (150).

In contrast to T cells, analyses of NK cell development in ADAP*^−/−^* mice revealed no significant alterations (151). Recently, we (48), and others (102), have extended these initial findings to provide a more detailed evaluation of NK cell functions in response to a variety of NK cell receptors. While *ADAP^−/−^* and WT NK cells demonstrated comparable cytotoxicity, we and others have observed attenuated cytokine production in *ADAP^−/−^* NK cells following activation *via* Ly49D (48, 102), NKG2D, NCR1, 2B4, and CD137 (4-1BB) (48). Thus, ADAP could act as a divergent point to selectively attenuate cytokine production without affecting cytotoxicity in NK cells.

## Future Perspectives

Identifying unique signaling myriads of signaling cascades in NK and T cells is complex, but improved understanding can serve to enhance existing immune-based therapies. For instance, new treatments that use genetically engineered lymphocytes to target malignancy have had successes in elimination of tumors such as refractory B cell malignancies, but have been limited by CRS. Targeting ADAP could help to regulate inflammatory cytokine production from effector lymphocytes while preserving cytotoxicity. Further, studies are also ongoing to test the efficacy of several pharmacological PI(3)K inhibitors in regulating tumor growth and survival without affecting the function of the immune cells. Continued detailed, rigorous study of lymphocyte signaling has the potential to unlock additional targets that could permit tailored responses for clinical therapy related to chronic autoimmunity and cancer.

## Author Contributions

KR, MR, CS, and SM wrote the major sections of the manuscript. SR, LW, and MT provided critical intellectual input and helped in writing. KR and SM compiled and revised the text; prepared the figures and the table.

## Conflict of Interest Statement

The authors declare that the research was conducted in the absence of any commercial or financial relationships that could be construed as a potential conflict of interest.
